# Delayed Presentation of Children to Healthcare Facilities due to COVID-19 Lockdown, Leading to Severe Complications

**DOI:** 10.5041/RMMJ.10431

**Published:** 2021-04-29

**Authors:** Yonatan Yeshayahu

**Affiliations:** Pediatrics Department, Samson Assuta Ashdod University Hospital, Goldman Medical School, Ben-Gurion University, Ashdod, Israel

**Keywords:** Children, complications, COVID-19, delayed diagnosis

## Abstract

During the coronavirus disease 2019 (COVID-19) pandemic, the increasing fear of leaving home and entering hospitals, together with guidelines to the public from Israel’s Ministry of Health recommending the use of telemedicine rather than physical visits to the doctor, led to delayed diagnoses of non-COVID-19-related medical conditions. This research letter presents a cluster of severe medical conditions that were delayed in diagnosis due to postponed presentation to healthcare facilities during the COVID-19 pandemic. Ewing sarcoma, severe hemolytic anemia, endocarditis requiring surgery, and septic hip requiring surgery are some examples of cases we encountered with delayed diagnoses. This led to the appearance of a rather low burden of disease in the pediatric population during the pandemic, and pediatric hospitals and clinics experienced a very low volume of activity. Given the low burden of COVID-19 in children, and the well-defined separation between infected and non-infected areas within the hospitals, we should consider improving the guidelines and messages conveyed to the public regarding the importance of prompt medical assessment for other medical conditions, even during a pandemic, along with reassurance of the safety of entering medical facilities given the strict isolation procedures being observed. Conclusion: Medical associations should reconsider the messages being sent to the public during future outbreaks, and encourage medical assessment.

## INTRODUCTION

During March–April 2020 the pandemic of coronavirus disease 2019 (COVID-19) led to a lockdown in many countries. Hospitals were focused on treatment of COVID-19 patients, and the public was encouraged not to go to hospitals and clinics unless absolutely essential.^1^ These public guidelines together with growing fear of leaving home and attending medical facilities led to a significant reduction of activity in pediatric departments and for community pediatricians.^2^–^4^ The avoidance of contact with medical facilities entailed a theoretic concern of delayed diagnosis of significant illnesses. An example of this concern was the recently published cluster of cases with delayed diagnoses of diabetic ketoacidosis.^5^

In this report we describe several different presentations of severe conditions in children who delayed presenting them to a doctor or complying with the recommended diagnostic workup.

## PATIENT MEDICAL REPORTS

### Endocarditis Leading to Cardiac Surgery

A 16-year-old boy with trisomy 21 and no additional medical condition, including no cardiac abnormalities, presented to the emergency room (ER) in early March 2020, with a 2-week fever accompanied by knee and hip pain. On examination a new heart murmur was detected, and lab tests revealed an elevated C-reactive protein (88 mg/L). The family was advised to admit their son for extended workup; however, they refused out of fear of being exposed to COVID-19 in the hospital and requested to continue workup in the community. Over the next seven weeks at home, the boy experienced recurrent fever and received two courses of amoxicillin prescribed by his family doctor due to positive group A streptococcus on throat culture. This resolved the fever; however, fever recurred when antibiotics were stopped. The patient was brought back to the hospital seven weeks after the previous visit, still with fever, a systolic murmur, and an elevated C-reactive protein (77 mg/L), thrombocytopenia of 79,000/μL, and a blood culture positive for Gram-positive cocci. Echocardiogram revealed signs of endocarditis with a large 2-cm vegetation on the mitral valve. Given insufficient response following four days of intravenous antibiotics, and given the large vegetation, the child underwent surgery for excision of the vegetation and reconstruction of the mitral valve.

### Severe Hemolytic Anemia

A 2.5-year-old boy with a history of tetralogy of Fallot, which had been fully corrected at the age of eight weeks, with no cardiac sequela, presented to the ER following two weeks of fatigue, irritability, recurrent chest and abdominal pain, low energy, and reduced appetite. During the previous week, he had dark urine and his parents noticed yellowish skin color. Only after observing these symptoms for two weeks did they bring him to the hospital. The boy was pale with a yellowish skin color, and blood tests were consistent with severe hemolytic anemia: hemoglobin, 4 g/dL; reticulocyte, 15.7%; lactate dehydrogenase, 870 U/L; and total bilirubin, 3.2 mg/dL, mostly indirect. Indirect Coombs test was positive. The boy was treated with packed cells, which temporarily increased his hemoglobin to 10 g/dL. However, the boy’s hemoglobin level declined again after several days to 7 g/dL, at which point steroid treatment was started.

### Severe Septic Hip

A 13-year-old boy presented to the ER following two weeks of right inguinal pain, which had become more severe with each passing day, first limping and eventually unable to bear weight. No fever was noted and no history suggesting rheumatic disease. On examination, pain on rotation of the hip joint was noted; he had a normal hip X-ray, normal complete blood count, and elevated C-reactive protein (250 mg/L). Ultrasound of the hip joint showed 8 mL of fluid collected in the joint, which was aspirated and consistent with septic arthritis; the aspirate contained 172,802 white blood cells with 93% polymorphonuclears and was culture-positive for methicillin-resistant *Staphylococcus aureus*. After two joint aspirations, treatment with vancomycin, and very slow improvement, magnetic resonance imaging was performed which showed extensive joint disease, involvement of the surrounding muscles with abscesses, and signs of osteomyelitis of the femur, acetabulum, and sacrum. The patient underwent surgery to drain an additional 20 mL of pus, drilling holes in the femur and ileum, and perform lavage.

### Ewing Sarcoma

A 4-year-old boy was brought to the ER following ten days of severe quadriparesis. The boy initially complained of fatigue and difficulty walking, which deteriorated within two days to complete inability to walk or use his arms. He received two medical con-sultations by telemedicine and received antibiotic treatment and systemic steroids. He remained at home, bedridden and spoon-fed for a full ten days before presenting to the ER. On admission he was noted to suffer severe proximal quadriparesis. Magnetic resonance imaging of the spine was performed demonstrating a space-occupying lesion along the spinal canal along C2–4, which measured 3.6×3.2× 3.2 cm. A biopsy was performed, returning the diagnosis of Ewing sarcoma. When the parents were consulted regarding the reason for delay in presenting to the hospital despite two weeks of significant neurological deterioration, their response was that they feared the COVID-19 pandemic and did not want to leave the house.

## DISCUSSION

These four cases all show a delayed diagnosis of serious conditions, with a severe disease upon presentation due to the delay. All parents reported the fear of leaving home and entering the hospital due to the COVID-19 pandemic as the reason for their delayed arrival and medical workup. During the lockdown in Israel, official guidelines dispersed by the Ministry of Health instructed people who felt unwell to stay at home and call an ambulance rather than arrive at a medical facility.^1^ Patients who had appointments with a pediatrician were notified that the visit would take place virtually using video or phone. The media covered the COVID-19 status in all hospitals, but did not stress enough the complete separation of coronavirus departments and ERs from other sections in the hospitals. Given the very low burden of disease which was seen in all pediatric departments worldwide,^3^–^5^ and the complete separation between COVID-19 patients and others, it is essential to reassess the message being sent to parents of children during this pandemic. Moving forward and looking towards the wave that may be experienced in the coming year, professional associations should consider disseminating a stronger message to families about the significant risk of delayed diagnoses of certain conditions, even during a large-scale pandemic, especially since the pediatric population has been relatively spared and pediatric departments saw small numbers of inpatients.

In summary, this report highlights the significant risk of developing severe medical conditions that are diagnosed late due to parental fear of going to the hospital, at a time when official messages from the Ministry of Health encourage patients to stay at home due to the COVID-19 pandemic.

## Abbreviations

COVID-19: coronavirus disease 2019
ER: emergency room.
